# A first description of chronic arthritis and uveitis in an adolescent girl with glycogen storage disease

**DOI:** 10.1186/1546-0096-10-S1-A93

**Published:** 2012-07-13

**Authors:** Karen N Watanabe Duffy, Robert Polomeno, Constantin Polychronakos, Ciaran M Duffy

**Affiliations:** 1 The Montreal Children's Hospital, McGill University Health Centre, Montreal, QC, Canada

## Purpose

Glycogen storage disease (GSD) type 1a is an autosomal recessive inborn error of carbohydrate metabolism which affects glycogenolysis and gluconeogenesis. Severe fasting hypoglycemia and secondary biochemical abnormalities such as hyperuricemia occur. Molecular analysis differentiates GSD type 1 into different clinical phenotypes, of which Type 1b but not Type 1a, is associated with inflammatory bowel disease (IBD). While gout has been described in GSD, chronic arthritis and uveitis have not.

## Methods

### Case report

A 15-year-old girl with GSD type 1a, presented with a 2 month history of right midfoot swelling. There were no systemic features and no history of previous infections. GSD type 1a had been diagnosed in infancy and genetic testing was positive for the 83R>C mutation. Her mother had inactive Crohn’s disease that had never required treatment. On musculoskeletal examination, she had a right midfoot arthritis but no enthesitis and back and sacroiliac joint examinations were normal. She had a mild microcytic anemia (Hgb 102 g/l) and an elevated erythrocyte sedimentation rate (ESR) of 30 mm (Winthrobe). No platelet dysfunction was identified. Serum uric acid was 559 (180-350 umol/L), which normalized within several months of strict dietary control. Serum glucose was normal and lactate was 6.2 (0-2.5 mmol/L). HLA-B27, rheumatoid factor (RF) and angiotensin converting enzyme were negative. The antinuclear antibody (ANA) was 1:80; extractable nuclear antigens (ENA), quantitative immunoglobulin and complement levels and urinalysis were normal. Abdominal ultrasound identified hepatic steatosis, as expected, without hepatomegaly or adenoma. X-rays of the right foot showed soft tissue swelling over the dorsum of the foot and the chest x-ray was normal. Subsequently, she developed right ankle and subtalar joint arthritis. The synovial fluid from the ankle was negative for both crystals and microorganisms. The right ankle, subtalar and midfoot joints were injected with triamcinolone hexacetonide, resulting in a short-lived response. Sulfasalazine was added since Methotrexate was contraindicated due to potential hepatotoxicity, compounded by the underlying disease. An MRI with gadolinium of the right ankle and foot confirmed synovitis and tenosynovitis with normal bone architecture and joint spaces. The patient then developed arthritis of the right wrist, left knee and left ankle. Nine months after the initial presentation, she developed anterior uveitis whose course was chronic and asymptomatic. Due to persisting disease activity and limited options for treatment due to the underlying disease, Adalimumab was initiated, which resulted in progressive and marked improvement of both the uveitis and the arthritis and was well-tolerated.

## Conclusion

This is the first description of a GSD patient with a chronic form of arthritis and uveitis. Synovial fluid analysis eliminated gout as a cause of arthritis. While IBD-related arthritis might have been postulated as the underlying cause, GSD type 1a is not associated with IBD. Of interest, treatment with Adalimumab in this patient resulted in significant resolution of both arthritis and uveitis.

## Disclosure

Karen N. Watanabe Duffy: None; Robert Polomeno: None; Constantin Polychronakos: None; Ciaran M. Duffy: None.

